# Erratum: miR-425-5p, a SOX2 target, regulates the expression of FOXJ3 and RAB31 and promotes the survival of GSCs

**DOI:** 10.26502/acbr.50170176

**Published:** 2021-06-15

**Authors:** Arlet María Acanda de la Rocha, Marisol González-Huarriz, Elizabeth Guruceaga, Nicole Mihelson, Sonia Tejada-Solís, Ricardo Díez-Valle, Naiara Martínez-Vélez, Juan Fueyo, Candelaria Gomez-Manzano, Marta M. Alonso, John Laterra, Hernando López-Bertoni

**Affiliations:** 1 The Health Research Institute of Navarra (IDISNA), USA; 2 Program in Solid Tumors and Biomarkers, Foundation for the Applied Medical Research, USA; 3 Department of Pediatrics, University Hospital of Navarra, Pamplona, Spain.; 4 Hugo W Moser Research Institute at Kennedy Krieger, Baltimore, MD, USA.; 5 Department of Neurology, The Johns Hopkins School of Medicine, Baltimore, MD, USA.; 6 Bioinformatics Unit, Center for Applied Medical Research, Pamplona, Spain.; 7 Department of Neurosurgery, University Hospital of Navarra, Pamplona, Spain.; 8 Department of Neuro-Oncology, The University of Texas MD Anderson Cancer Center, Houston, Texas, USA; 9 Department of Genetics, The University of Texas MD Anderson Cancer Center, Houston, Texas, USA; 10 Department of Neuroscience, The Johns Hopkins School of Medicine, Baltimore, MD, USA; 11 Department of Oncology, The Johns Hopkins School of Medicine, Baltimore, MD, USA.; 12 Department of Environmental Health Sciences. Robert Stempel College of Public Health & Social Work. Florida International University, USA.

## ERRATUM:

After publication of the original article, the authors noted that the following error had occurred:

The wrong publication was referenced in the analysis performed to identify Sox2-regulated miRNAs in the section entitled “**Molecular profiling identified a subset of SOX2-regulated miRNAs in glioma stem cells”.** In the passage that reads:

To provide robustness to this miRNA profile, we cross-referenced our miRNA array results to a set of miRNAs identified by Lopez-Bertoni and colleagues to be regulated by exogenous expression of SOX2 in GSCs (Lopez-Bertoni et al. 2015). Lopez et. al. identified 21 down-regulated and 56 up-regulated miRNAs in GSCs expressing exogenous SOX2.

